# The Bicycle from a Surgical Standpoint

**Published:** 1895-09

**Authors:** 


### The Bicycle from a Surgical Standpoint.

From the medical point of view, this subject has chiefly been discussed in connection with the effects of over-exertion. The latter are well-known, are observed in every form of outdoor exercise, and form, with the greatest propriety, a text for warnings the neglect of which may bring about, in some instances, quite serious results.

As surgeons, the important question to us is whether bicycling has a tendency to bring about any peculiar bodily malformations due to its use, whether moderate or excessive and to cause any distinct surgical diseases.

In the very first place we must speak of the leaning over indulged in, to a greater or less degree, by the majority of wheelmen. It would necessitate the printing of many and corpulent volumes to produce all the exaggerated and nonsensical statements that have been published in reference thereto. As a matter of fact, the writer, whose experience with bicycles dates back nearly a quarter of a century, is convinced that an absolutely erect position of the body, while riding a bicycle, is a mistaken one and is fraught with possibilities for harm. Sitting absolutely straight in a bicycle saddle tends to throw all the weight upon the rider's seat, whereas a certain amount of leaning forward distributes the weight more evenly, as the shoulders then bear an appreciable amount of the burden. A moderate amount of bending forward facilitates the steering, gives better play to the pvll of the arms upon the handles and allows of greater force being applied to the pedals. An exaggerated leaning forward may certainly produce a gradual vertebral displacement, and is decidedly ungraceful.

If a race meet is attended, and the interested looker-on closely observes the athletic contestants, he will certainly notice that they are as straight and well set-up a lot of young men as can usually be seen, and that their bent positions appear to be entirely reserved for the actual riding time. Now that nearly every young man, and a good many old ones, ride, we certainly occasionally see cases of spinal difficulties in bicyclists, but we will have to use caution in asserting that the wheel itself has been at fault. A hump backed gentleman of our acquaintance, who rides a wheel and enjoys it greatly, told us the other day that on several occasions he had been asked if the wheel had not "done it," and that he has been pointed out as an awful example. But as his hump dates back from his nursing days he continues his riding.

Upon somewhat better ground attention has been called to the possibilties of rectal or prostatic troubles due to bicycle riding. The writer feels justified in saying that such troubles rarely occur as a direct result of wheling. Hemorrhoidal troubles are so nearly universal in men that caution is needed in ascribing their occurrences to the use of the bicycle in every instance in which they happen in a rider. It is easily conceivable that if the saddle, or the clothes worn by the rider, chafe or irritate the perianal region in an individual whose digestive organs are not in good order, an attack of piles may occur.

As for prostatic troubles, we have been assured that some have been as direct results of riding. In our long experience of wheelling we have failed to see any cases, but there is good reason to believe that caution is necessary in advising male adults to ride, and that the existence of prostatic trouble should be a signal for giving up the wheel.—*Int. Journal of Surgery.*

